# Assessment of the effect of culture components of clinical *Pseudomonas aeruginosa* isolates on vancomycin eradication of *Staphylococcus aureus* mature biofilms

**DOI:** 10.1186/s13104-026-07718-5

**Published:** 2026-03-10

**Authors:** Mona Farag, Abeer Ghazal, Gamaleldin Elsawaf, Ghada Abu-Sheasha, Aliaa Aboulela

**Affiliations:** 1 Medical Research Institute, Microbiology Department, Alexandria University, 165 El-Horreya Avenue, Al-Ibrahimeya, Alexandria, Egypt; 2 Medical Research Institute, Biomedical Informatics and Medical Statistics Department, Alexandria University, Alexandria, Egypt

**Keywords:** Antibiofilm, Methicillin-Resistant *Staphylococcus aureus* biofilm, Multidrug resistant *Pseudomonas aeruginosa*, Vancomycin, Superinfection, Gene expression

## Abstract

**Background:**

*P. aeruginosa* and *S. aureus* are frequently co-isolated from chronic biofilm-associated infections. Microbial interactions within biofilms affect antimicrobial susceptibility, making treatment more challenging. This study aimed to investigate the effect of *P. aeruginosa* superinfection on mature *S. aureus* biofilm eradication by vancomycin.

**Methods:**

Biofilm-producing clinical isolates of *S. aureus* and MDR *P. aeruginosa* were collected from chronic wound infections. Baseline viable cell counts in 24-hour *S. aureus* biofilms were determined. The minimal biofilm eradication concentration (MBEC) of vancomycin was assessed in the presence and absence of *P. aeruginosa* culture components. Relative gene expression of *ldh* and *adh* in biofilm cells was evaluated by RT-qPCR.

**Results:**

Superinfection with *P. aeruginosa* reduced the viable counts of *S. aureus* biofilm cells and altered vancomycin MBEC for planktonic-phase biofilm cells. Disruption of *S. aureus* biofilms by MDR *P. aeruginosa* culture supernatants reduced *ldh* and *adh* gene expression, with an effect comparable to, but not additive with, that of vancomycin.

**Conclusion:**

Vancomycin eradication of *S. aureus* planktonic biofilm cells is altered by MDR *P. aeruginosa* superinfection. Both vancomycin and MDR *P. aeruginosa* culture supernatants, individually and combined, interfered with the anaerobic respiration pathways in sessile biofilm cells.

**Supplementary Information:**

The online version contains supplementary material available at 10.1186/s13104-026-07718-5.

## Introduction

Pathogenic bacteria often form biofilms on chronic wounds. Biofilms consist of sessile microbial aggregates embedded in a self-produced extracellular polymeric matrix, exhibiting distinct growth and gene expression phenotypes compared to planktonic cells [1]. Eliminating *Staphylococcus aureus* biofilms is challenging, particularly methicillin-resistant strains (MRSA), for which vancomycin is the standard treatment [2]. In vitro studies show that high vancomycin concentrations can penetrate *S. aureus* biofilms and reduce viable cells [3], though penetration is often slow and incomplete [4, 5]. Eradication requires concentrations over 1000 times the Minimal inhibitory concentration (MIC) [2, 6]. Microbial interactions in biofilm-related infections complicate treatment [7].

*Staphylococcus aureus* and *Pseudomonas aeruginosa* frequently co-exist in persistent infections [8, 9], with complex interspecies interactions [10, 11]. *P. aeruginosa* outcompetes other bacteria via cell-associated and secreted virulence factors. Some extracellular products of *P. aeruginosa* disturb electrons flow through the respiratory chain of *S. aureus* creating a state of oxidative stress. This induces upregulation of dehydrogenase enzymes, such as lactate dehydrogenase (Ldh), and aldehyde-alcohol dehydrogenase (Adh), which promote switching of *S. aureus* from aerobic respiration to lactic acid fermentation [9, 12–15]. The cumulative effect of *P. aeruginosa* extracellular factors includes reduced viability, enhanced biofilm formation, and induction of the small colony variants (SCVs) [12, 16]. The combined impact of *P. aeruginosa* supernatants and antimicrobials on mature *S. aureus* biofilms remains debated [17]. This study aimed to evaluate how multidrug-resistant (MDR) *P. aeruginosa* superinfection affects vancomycin’s efficacy against mature *S. aureus* biofilms.

## Materials and methods

The study included 5 *P. aeruginosa* and 10 *S. aureus*: 5 Methicillin Resistant *S. aureus* (MRSA), and 5 Methicillin Susceptible *S. aureus* (MSSA) isolates from chronic leg ulcers submitted to the Microbiology Laboratory, Medical Research Institute, Alexandria University, Egypt. Identification and susceptibility testing were done using the BD Phoenix^™^ M50 System. All *S. aureus* isolates were vancomycin-susceptible (Figs. S1 and S2), while all *P. aeruginosa* isolates were multidrug resistant (Fig. S3).

### *S. aureus* mature biofilm formation and viable cell count

*S. aureus* suspensions (10⁵ CFU/ml) in Muller Hinton Broth (MHB) with 1% glucose were added to sterile 96-well plates and incubated at 37 °C for 24 h. Biofilm formation and assessment of biofilm strength were assessed by crystal violet staining and spectrophotometry as described by Stepanović et al. [18]. Planktonic phases were discarded, wells washed, and 100 µl saline was added to resuspend sessile cells, which were detached by scraping [19]. Viable cell counts were determined by quantitative culture on mannitol salt agar (MSA) plates, as previously described [20].

### Preparation of *P. aeruginosa*-derived biofilm disrupting agents and activity testing

*Cell-free culture supernatants: P. aeruginosa* was cultured in MHB at 37 °C with shaking (225 rpm) for ~ 24 h, adjusted to 0.5 McFarland (1.5 × 10^8^ CFU/ml), centrifuged at 10,000 rpm for 10 min. Supernatants were filter-sterilized, verified for sterility on blood agar, and stored at − 20 °C [21].

*Cell suspensions:* Fresh colonies of *P. aeruginosa* from overnight blood agar cultures were adjusted in MHB to 0.5 McFarland for each experiment.

*Biofilm disruption:* Mature *S. aureus* biofilms (10 isolates) were prepared as described above. After 24 h, planktonic cells were removed and wells washed. Cells and sterile supernatant of one randomly selected *P. aeruginosa* isolate (P1) were added separately. Fresh media was added to controls. Plates were re-incubated at 37 °C for 24 h then planktonic cells were removed, and wells were washed. Sessile cells were scraped, suspended in 0.9% saline, vortexed, serially diluted, and cultured on MSA plates. For each isolate, the experiment was done in triplicate in the same settings.

*Statistical analysis:* Viable cell reduction was quantified as the log_10_ reduction in CFU/mL, calculated as the difference between log_10_-transformed baseline and post-treatment values. Mean log_10_ reductions with 95% confidence intervals (CI) were estimated for MRSA and MSSA isolates after exposure to P1 cell suspension and cell-free supernatant using linear mixed-effects models with treatment as a fixed effect and isolate as a random intercept. Pre-specified contrasts were evaluated relative to no reduction (log_10_ reduction = 0) and between treatments, with Holm adjustment.

### Determination of minimal biofilm eradication concentration (MBEC) of Vancomycin and the modulatory effects of *P. aeruginosa* culture components

Six *S. aureus* isolates (3 MRSA, 3 MSSA) were randomly selected for MBEC testing. Serial 2-fold vancomycin dilutions (4096–2 µg/ml) were prepared in MHB with 1% glucose. Mature biofilms were formed in Microtitre plates (MTPs). After discarding planktonic cells and washing, vancomycin was added. Plates were incubated for 24 h. Baseline biofilms and negative controls were included. After incubation, planktonic cells were collected prior to rinsing and biofilm cell collection. MBECs for planktonic and sessile cells were determined using the drop plate method on MSA, as described by Naghili et al. [22]. The concentration of vancomycin in the well corresponding to no growth of *S. aureus* on MSA plates was considered as the MBEC.

To determine the modulatory effect of *P. aeruginosa* culture components on vancomycin MBEC against *S. aureus* biofilms, vancomycin MBECs against the 6 *S. aureus* isolates were re-evaluated with *P. aeruginosa* culture components. Mature biofilms were prepared, planktonic phases removed, and wells filled with *P. aeruginosa* cells or supernatant. Vancomycin was serially 2-fold diluted in the wells using each of *P. aeruginosa* culture components as the diluent; MHB alone was used for controls. Plates were incubated for 24 h, and MBECs were redetermined for each of the planktonic and sessile cells and compared to those with vancomycin alone.

*Statistical analysis:* Vancomycin MBEC values for planktonic-phase *S. aureus* biofilm cells were log-transformed and analyzed separately using mixed-effects models to compare baseline, cell-free supernatant, and cell suspension conditions, accounting for repeated measurements and multiple *P. aeruginosa* preparations. Holm adjustment was applied to pairwise comparisons.

### Determination of the effect of *P. aeruginosa* culture supernatants and Vancomycin on *ldh* and *adh* gene expression in *S. aureus* sessile biofilm cells

To assess the impact of vancomycin and *P. aeruginosa* supernatants, alone and combined, on *ldh* and *adh* gene expression in sessile biofilm cells, MRSA 21 and *P. aeruginosa* strains P1, P2, and P4 were selected based on their differential effects on vancomycin MBEC. Primers for *ldh* and *adh* were designed using NCBI Primer-BLAST. 16 S rRNA was used as the housekeeping gene [23]. Primer sequences are listed in Table S1.

*RNA extraction and cDNA synthesis:* Mature MRSA 21 biofilms were treated with *P. aeruginosa* supernatants, ½ sub-MBEC vancomycin, and their combinations. After incubation, planktonic cells were removed, the wells were rinsed, and sessile cells were scraped and resuspended in 100 µL RNAzol^®^ RT. Lysis was enhanced by three freeze-thaw cycles and syringe disruption (27-gauge needle). RNA was extracted following the RNAzol^®^ RT protocol, and cDNA was synthesized from 1 µg/ml RNA using the Thermo Scientific RevertAid First Strand cDNA Synthesis Kit.

*Quantitative real-time PCR* reactions were run in quadruplicate using a 20 µL mix: 10 µL Maxima SYBR Green Master Mix, 0.5 µL each primer, and 2 µL cDNA. PCR was performed on a Stratagene Mx3000P system with thermal cycling at 95 °C for 10 min, followed by 40 cycles of 95 °C for 15 s, 52 °C for 30 s, and 72 °C for 30 s. Fold changes in *adh*, and *ldh* gene expression were calculated using the comparative Ct (2⁻ΔΔCt) method and reported with 95% confidence intervals; confidence intervals crossing a fold change of 1 were considered not statistically significant.

*Statistical analysis:* All statistical analyses were performed using R version 4.2.2 (R Foundation for Statistical Computing, Vienna, Austria).

## Results

All MRSA strains were moderate biofilm formers. Among MSSA isolates, 60% were strong and 40% moderate biofilm formers. Viable sessile cell counts in MRSA biofilms ranged from 1.35 × 10^8^ to 3.2 × 10^8^ CFU/mL (Table S2), while MSSA counts ranged from 3.5 × 10^8^ to 2.5 × 10^9^ CFU/mL (Table S3).

**Fig. 1:**
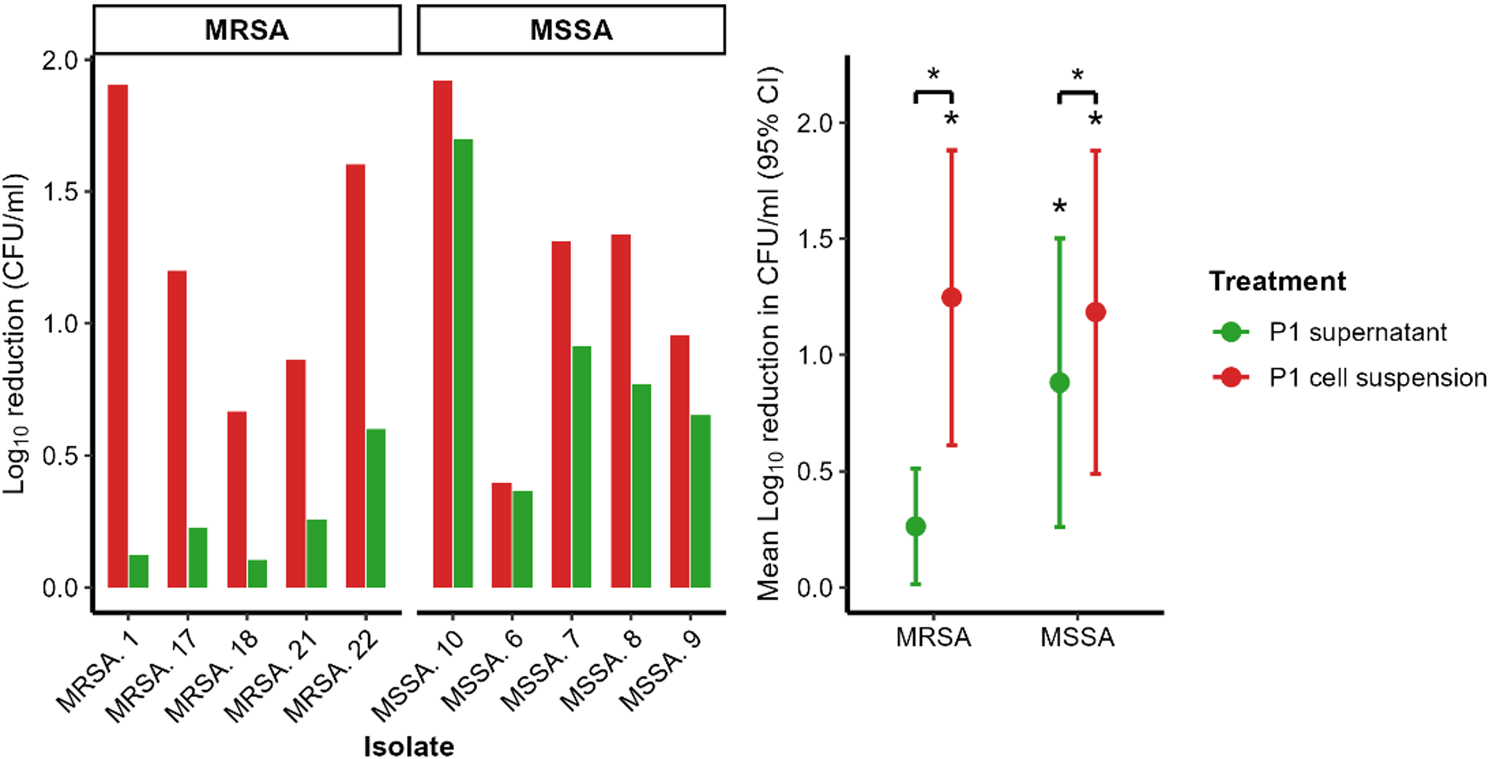
Log_10_ reduction (CFU/ml) of sessile *S. aureus* biofilm cells following interaction with P1 cell-free culture supernatant and P1 cell suspension, shown for individual isolates (left) and as group means with 95% confidence intervals (right). Asterisks above error bars indicate statistically significant differences from no reduction (log_10_ reduction = 0); horizontal brackets indicate pairwise comparisons between P1 cell suspension and cell-free supernatant within bacterial group, evaluated using linear mixed-effects models with Holm adjustment

**The cell suspension** of P1 resulted in a significant reduction in the viable cell counts of the sessile phases of mature biofilms formed by the 5 MRSA (*p*= 0.0002) and the 5 MSSA isolates (*p*= 0.0125) compared to the baseline. Among MRSA, log_10_ reductions ranged from 0.67 to 1.90, corresponding to minimal to low–moderate antibacterial activity. MSSA log_10_ reductions ranged from 0.40 to 1.92 (Fig. 1, Tables S2 and S3).

**The cell-free culture supernatant** of P1 induced smaller reductions in sessile biofilm viability, which was statistically significant only with MSSA (*p*= 0.0181). Log_10_ reductions among MRSA isolates ranged from 0.1 to 0.6, whereas MSSA Log_10_ reductions ranged from 0.37 to 1.7 (Fig. 1, Tables S2 and S3).

Overall, the cell suspension demonstrated stronger antibiofilm effects than the corresponding supernatant across all tested isolates. A statistically significant difference was observed between the effect of P1 cell suspension and its cell-free culture supernatant on MRSA (*p* = 0.0108) and MSSA isolates (*p* = 0.0272) (Fig. 1).

**Fig. 2:**
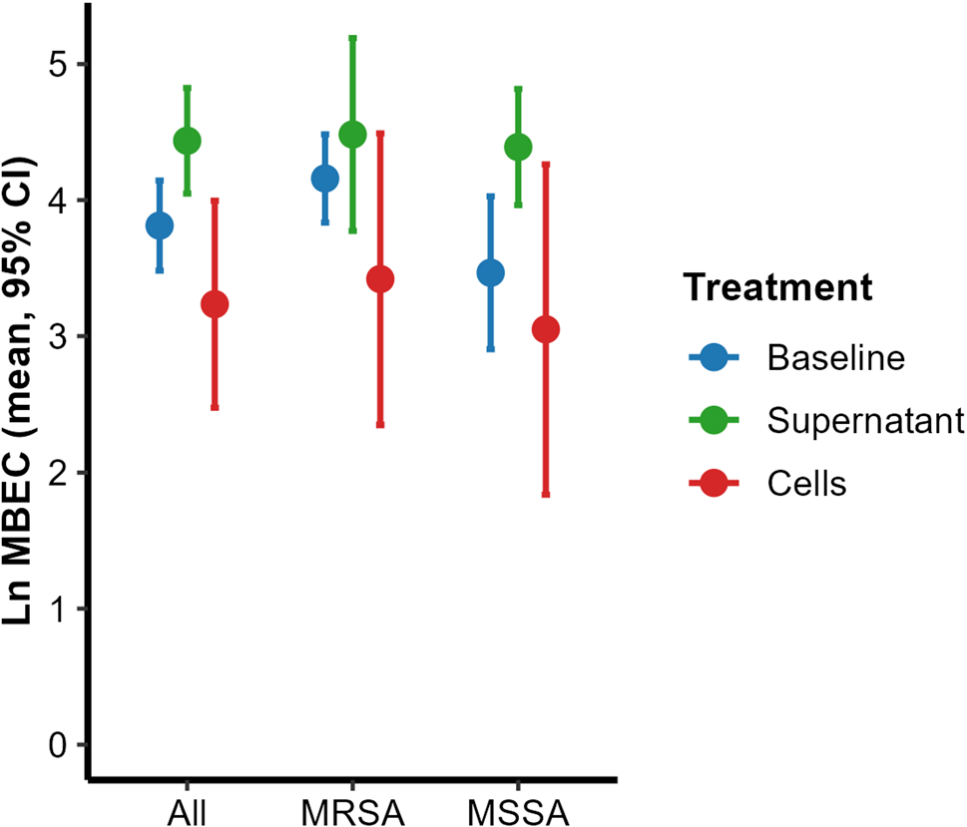
Effect of *P. aeruginosa*–derived biofilm-disrupting agents on vancomycin MBEC. The mean vancomycin MBEC for *S. aureus* cells in the planktonic phases of biofilms at baseline and after interaction with the supernatants and cell suspensions of five clinical MDR *P. aeruginosa* isolates. Ln MBEC: natural logarithm of MBEC values of vancomycin (in µg/ml) for *S. aureus* isolates. CI: confidence interval. All: combined results for MRSA and MSSA isolates

**Baseline MBEC of vancomycin** for the 6 isolates of *S. aureus* ranged from 16 to 128 µg/ml for the planktonic phase cells, and it was 4096 µg/ml or higher for the sessile phase cells (Fig. 2, Figs S4 and S5, Table S4). The overall changes in vancomycin MBEC induced by the cells and the supernatant across all isolates were not statistically significant after adjustment for multiple comparisons (adjusted *p* = 0.092, 0.069, respectively), (Table S9). *P. aeruginosa* cells superinfection mostly induced a reduction in vancomycin MBEC against MRSA and MSSA cells at the planktonic phase of mature biofilm, which was more observed with MRSA biofilms (Fig. 2, Fig. S4, Tables S4-S9). *P. aeruginosa* cell-free culture supernatants mostly induced an increase in the vancomycin MBEC against MRSA and MSSA planktonic cells. This increase was more evident with MSSA than MRSA (Table S9). No clear effect was observed on sessile *S. aureus* cells, as MBEC values before and after exposure to *P. aeruginosa* components exceeded the assay’s maximum vancomycin concentration (4096 µg/ml).

**Fig. 3:**
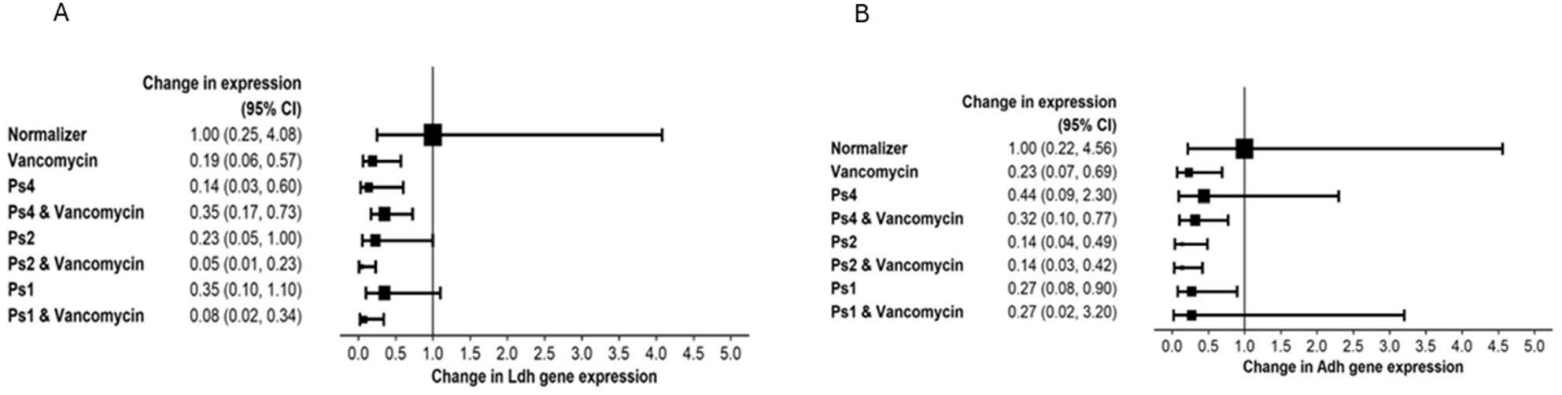
Forest plot chart for *ldh* (**A**) and *adh* (**B**) relative gene expression in MRSA 21 sessile biofilm cells at baseline and after disruption with vancomycin and/or *P. aeruginosa*-derived culture supernatants. A minimum and maximum interval including the null value of 1: the net effect of interaction cannot be determined. A whole interval below 1: the net effect of interaction is down regulation. A whole interval above 1: the net effect of interaction is up regulation. Ps4, Ps2, Ps1: identifiers for the cell free culture supernatants from *P. aeruginosa* strains P4, P2, and P1

***P. aeruginosa*** **cell-free culture supernatant and vancomycin**,** alone and combined**, caused down regulation in the expression of the key fermentative-pathway genes (*ldh* and *adh*) in the sessile cells of MRSA21 mature 24-hour biofilm (Fig. 3).

## Discussion

Biofilms involving *S. aureus* and *P. aeruginosa* on chronic wounds make treatment difficult. Studies have shown these pathogens may coexist competitively or cooperatively [14, 24–26]. Their complex interaction has been widely studied [12, 17, 26]. Conflicting results and variable conditions call for further research, especially with multidrug resistance impacting treatment decisions. This study examined how MDR *P. aeruginosa* superinfection affects vancomycin’s ability to eradicate mature *S. aureus* biofilms, using clinical isolates from chronic wound infections.

Mature biofilms are harder to eradicate than early-stage ones. Most studies assessed *P. aeruginosa*’s impact on *S. aureus* in co-cultured developing biofilms [12, 13, 15]. Trizna et al. reported higher *S. aureus* viability in established biofilms exposed to *P. aeruginosa* superinfection compared to co-cultures [11]. This work showed that superinfection of mature *S. aureus* biofilms with MDR *P. aeruginosa* significantly reduced MRSA and MSSA cell counts (*p* = 0.0002 and 0.0125). These findings align with Woods et al. and Reigada et al., who also showed reduced *S. aureus* viability after superinfection [21, 27].

*P. aeruginosa* cell-free supernatant contains various virulence factors, with HQNO and siderophores most linked to its effect on *S. aureus* survival and drug susceptibility in dual-species biofilms [12, 15, 17, 28, 29]. MDR strains may produce different profiles, contributing to more severe infections [30–32].

We assessed the impact of MDR *P. aeruginosa* supernatant on *S. aureus* biofilms. It reduced viable **sessile cells**, significantly for MSSA (*p* = 0.0181), but not MRSA (*p* = 0.1688). These findings align with Hoffman et al., who attributed growth inhibition to HQNO [33]. The weaker MRSA response observed may reflect acquisition of resistance traits enhancing its competitiveness in multispecies settings.

Our results show that MDR *P. aeruginosa* cells reduce sessile *S. aureus* (MRSA and MSSA) viability more than the cell-free supernatant, likely due to highly active cell-associated virulence factors like secretion systems.

Previous studies showed *P. aeruginosa* can alter *S. aureus* susceptibility to antibiotics and antiseptics [8, 17]. Orazi et al. reported that unlike the cells, the supernatant alone increased vancomycin tolerance in *S. aureus* Newman strain [12]. We observed similar results: combining supernatant with vancomycin raised MBEC for planktonic MRSA and MSSA, though responses varied by strain (Fig. S4, Tables S4–S8). This variability suggests that supernatant components alone do not dictate the outcome; *S. aureus* strains likely have intrinsic, strain-specific responses to *P. aeruginosa* secreted products.

The protective effect of *P. aeruginosa* supernatant against vancomycin is mainly attributed to HQNO and siderophores, which induce a metabolic shift toward fermentation and lactate production [8, 12, 33]. In contrast, LasA endopeptidase enhances vancomycin action [17]. Variability in exoproduct levels among *P. aeruginosa* isolates explains the differing supernatant effects.

In this study, contrary to the readily measurable effects in **planktonic phases** of biofilms, the effects of *P. aeruginosa* supernatants on vancomycin MBEC could not be assessed in **sessile phases** of *S. aureus* biofilms, as the highest tested concentration (4096 µg/ml) had minimal impact on viability. This concentration eradicated sessile cells in only 3/6 isolates (2 MSSA, 1 MRSA), but none when combined with supernatant.

Unlike the supernatant, co-treatment with *P. aeruginosa* cells and vancomycin lowered the MBEC for **planktonic-phase** MRSA, however, the change did not reach statistical significance (*p*= 0.092). Given that wound colonization usually starts with *S. aureus* and is often followed by secondary *P. aeruginosa* infection (gram-negative shift) [34, 35], these findings suggest that in superinfection, vancomycin may be more effective when administered early. Superinfection differs from coculture, where both species are introduced simultaneously. In coculture, *P. aeruginosa* shields *S. aureus* from vancomycin by pushing it deeper into the biofilm [11]. In mature dual-species biofilms, *S. aureus* becomes more invasive due to synergistic growth [24], highlighting the need for early antimicrobial treatment.

In **sessile biofilm phases**, *P. aeruginosa* promotes anaerobic respiration in *S. aureus* via upregulation of fermentation-related dehydrogenase genes [15, 36], aiding adaptation to hypoxia [37]. We measured *ldh* and *adh* expression in *S. aureus* **sessile cells** after treatment with vancomycin and MDR *P. aeruginosa* supernatants. Both genes were consistently downregulated by all agents; vancomycin plus supernatant showed no additive effect.

Expression of *ldh* and *adh* is vital for biofilm microcolony formation; their downregulation promotes sessile cell dispersal [38, 39]. Our results suggest that overnight exposure to vancomycin and/or MDR *P. aeruginosa* supernatants interfere with *S. aureus* adaptive survival pathways under hypoxia, promoting their dispersal to the planktonic phase, where they are more vancomycin sensitive. This supports Post et al., who reported that vancomycin’s antibiofilm action is time dependent [3]. Thus, vancomycin or MDR *P. aeruginosa* products may induce metabolic stress in *S. aureus* biofilm cells via reduced *ldh* and *adh* expression. Our findings show vancomycin’s effect on *S. aureus* requires prolonged exposure (up to 24 h).

Additional studies on a wider scale are needed to confirm the effect of vancomycin and *P. aeruginosa* supernatants on the metabolic pathways and survival mechanisms in *S. aureus* biofilm cells. Future research should also explore the potential of using well-characterized supernatants or isolated components, such as LasA endopeptidase, as adjuvant therapies for eradicating mature *S. aureus* biofilms due to their staphylolytic activity.

## Limitations

- Component analysis of *P. aeruginosa* cell-free supernatants was not performed due to lack of reference materials and equipment.
- The number of isolates included was limited due to the time-intensive and experimentally demanding nature of the workflow.
- Due to financial constraints, gene expression analysis was limited to one MRSA isolate and supernatants from three *P. aeruginosa* isolates.

## Supplementary Information

Below is the link to the electronic supplementary material.

Supplementary Material 1.

## Data Availability

Data generated or analyzed during this study are included in this published article and its supplementary information file.

## References

[1] Liu Y, Long S, Wang H, Wang Y. Biofilm therapy for chronic wounds. Int Wound J. 2024;21(2):e14667. 10.1128/cmr.15.2.167-193.2002. 38339793 10.1111/iwj.14667 PMC10858329

[2] Yee R, Yuan Y, Tarff A, Brayton C, Gour N, Feng J, Zhang Y. Eradication of Staphylococcus aureus biofilm infection by persister drug combination. Antibiot (Basel). 2022;11(10):1278. 10.3390/antibiotics11101278. 10.3390/antibiotics11101278 PMC9598165 36289936

[3] Post V, Wahl P, Richards RG, Moriarty TF. Vancomycin displays time-dependent eradication of mature *Staphylococcus aureus* biofilms. J Orthop Res. 2017;35(2):381–8. 10.1002/jor.23291. 27175462 10.1002/jor.23291

[4] Jefferson KK, Goldmann DA, Pier GB. Use of confocal microscopy to analyze the rate of Vancomycin penetration through *Staphylococcus aureus* biofilms. Antimicrob Agents Chemother. 2005;49(6):2467–73. 10.1128/AAC.49.6.2467-2473.2005. 15917548 10.1128/AAC.49.6.2467-2473.2005 PMC1140491

[5] Kaneko H, Nakaminami H, Ozawa K, Wajima T, Noguchi N. In vitro anti-biofilm effect of anti-methicillin-resistant *Staphylococcus aureus* (anti-MRSA) agents against the USA300 clone. J Glob Antimicrob Resist. 2021;24:63–71. 10.1016/j.jgar.2020.11.026. 33307275 10.1016/j.jgar.2020.11.026

[6] Rivani E, Arfijanto MV, Widodo ADW. Vancomycin for methicillin-resistant *Staphylococcus aureus* biofilm eradication is associated with the emergence of heterogeneous Vancomycin intermediate *Staphylococcus aureus*. Int J Health Sci. 2022;6(S9):811–8. 10.53730/ijhs.v6nS9.12536.

[7] Coenye T. Biofilm antimicrobial susceptibility testing: where are we and where could we be going? Clin Microbiol Rev. 2023;36(4):e00024–00023. 10.1128/cmr.00024-23. 37812003 10.1128/cmr.00024-23 PMC10732061

[8] Orazi G, Ruoff KL, O’Toole GA. *Pseudomonas aeruginosa* increases the sensitivity of biofilm-grown *Staphylococcus aureus* to membrane-targeting antiseptics and antibiotics. MBio. 2019. 10.1128/mBio.01501-19. 10.1128/mBio.01501-19 PMC6667622 31363032

[9] Yung DBY, Sircombe KJ, Pletzer D. Friends or enemies? The complicated relationship between *Pseudomonas aeruginosa* and *Staphylococcus aureus*. Mol Microbiol. 2021;116(1):1–15. 10.1111/mmi.14699. Epub 2021 Mar 8. 33576132 10.1111/mmi.14699

[10] Biswas L, Götz F. Molecular mechanisms of *Staphylococcus* and *Pseudomonas* interactions in cystic fibrosis. Front Cell Infect Microbiol. 2022;11:824042. 10.3389/fcimb.2021.824042. 35071057 10.3389/fcimb.2021.824042 PMC8770549

[11] Trizna EY, Yarullina MN, Baidamshina DR, Mironova AV, Akhatova FS, Rozhina EV, et al. Bidirectional alterations in antibiotics susceptibility in *Staphylococcus aureus*—*Pseudomonas aeruginosa* dual-species biofilm. Sci Rep. 2020;10(1):14849. 10.1038/s41598-020-71834-w. 32908166 10.1038/s41598-020-71834-w PMC7481796

[12] Orazi G, O’Toole GA. Pseudomonas aeruginosa alters Staphylococcus aureus sensitivity to Vancomycin in a biofilm model of cystic fibrosis infection. MBio. 2017;8(4):mbio10112800873–17. 10.1128/mBio.00873-17. 10.1128/mBio.00873-17 PMC5516255 28720732

[13] Mashburn LM, Jett AM, Akins DR, Whiteley M. *Staphylococcus aureus* serves as an iron source for *Pseudomonas aeruginosa* during in vivo coculture. J Bacterial. 2005;187(2):554–66. 10.1128/JB.187.2.554-566.2005. 10.1128/JB.187.2.554-566.2005 PMC543556 15629927

[14] DeLeon S, Clinton A, Fowler H, Everett J, Horswill AR, Rumbaugh KP. Synergistic interactions of *Pseudomonas aeruginosa* and *Staphylococcus aureus* in an in vitro wound model. Infect Immun. 2014;82(11):4718–28. 10.1128/IAI.02198-14. 25156721 10.1128/IAI.02198-14 PMC4249327

[15] Filkins LM, Graber JA, Olson DG, Dolben EL, Lynd LR, Bhuju S, O’Toole GA. Coculture of *Staphylococcus aureus* with *Pseudomonas aeruginosa* drives *S. aureus* towards fermentative metabolism and reduced viability in a cystic fibrosis model. J Bacteriol. 2015;197(14):2252–64. 10.1128/JB.00059-15. 25917910 10.1128/JB.00059-15 PMC4524177

[16] Mitchell G, Séguin DL, Asselin AE, Déziel E, Cantin AM, Frost EH et al. *Staphylococcus aureus* Sigma B-dependent emergence of small-colony variants and biofilm production following exposure to *Pseudomonas aeruginosa* 4-hydroxy-2-heptylquinoline-N-oxide.BMC microbiol. 2010;10:1–15. 10.1186/1471-2180-10-33. 10.1186/1471-2180-10-33 PMC2824698 20113519

[17] Radlinski L, Rowe SE, Kartchner LB, Maile R, Cairns BA, Vitko NP, et al. *Pseudomonas aeruginosa* exoproducts determine antibiotic efficacy against *Staphylococcus aureus*. PLoS Biol. 2017;15(11):e2003981. 10.1371/journal.pbio.2003981. 29176757 10.1371/journal.pbio.2003981 PMC5720819

[18] Stepanović S, Vuković D, Hola V, Bonaventura GD, Djukić S, Ćirković I, et al. Quantification of biofilm in microtiter plates: overview of testing conditions and practical recommendations for assessment of biofilm production by Staphylococci. Apmis. 2007;115(8):891–9. 10.1111/j.1600-0463.2007.apm_630.x. 17696944 10.1111/j.1600-0463.2007.apm_630.x

[19] Kragh KN, Alhede M, Kvich L, Bjarnsholt T. Into the well—a close look at the complex structures of a microtiter biofilm and the crystal Violet assay. Biofilm. 2019;1:100006. 10.1016/j.bioflm.2019.100006. 33447793 10.1016/j.bioflm.2019.100006 PMC7798451

[20] Wilson C, Lukowicz R, Merchant S, Valquier-Flynn H, Caballero J, Sandoval J, Okuom M, Huber C, Brooks TD, Wilson E, Clement B, Wentworth CD, Holmes AE. Quantitative and Qualitative Assessment Methods for Biofilm Growth: A Mini-review. Res Rev J Eng Technol. 2017. http://www.rroij.com/open-access/quantitative-and-qualitative-assessment-methods-for-biofilm-growth-a-minireview-.pdf. Epub 2017 Oct 24. PMID: 30214915; PMCID: PMC6133255. PMC6133255 30214915

[21] Woods PW, Haynes ZM, Mina EG, Marques CN. Maintenance of *S. aureus* in co-culture with *P. aeruginosa* while growing as biofilms. Front Microbiol. 2019;9:3291. 10.3389/fmicb.2018.03291. 30687276 10.3389/fmicb.2018.03291 PMC6333908

[22] Naghili H, Tajik H, Mardani K, Razavi Rouhani SM, Ehsani A, Zare P. Validation of drop plate technique for bacterial enumeration by parametric and nonparametric tests. Vet Res Forum. 2013;4(3):179–83. PMID: 25653794; PMCID: PMC4312378. 25653794 PMC4312378

[23] Atarashi K, Tanoue T, Shima T, Imaoka A, Kuwahara T, Momose Y, et al. Induction of colonic regulatory T cells by Indigenous *Clostridium* species. Science. 2011;331(6015):337–41. 10.1126/science.1198469. 21205640 10.1126/science.1198469 PMC3969237

[24] Pastar I, Nusbaum AG, Gil J, Patel SB, Chen J, Valdes J, Stojadinovic O, Plano LR, Tomic-Canic M, Davis SC. Interactions of methicillin resistant *Staphylococcus aureus* USA300 and *Pseudomonas aeruginosa* in polymicrobial wound infection. PLoS ONE. 2013;8(2):e56846. 10.1371/journal.pone.0056846. 23451098 10.1371/journal.pone.0056846 PMC3579943

[25] Serra R, Grande R, Butrico L, Rossi A, Settimio UF, Caroleo B, et al. Chronic wound infections: the role of *Pseudomonas aeruginosa* and *Staphylococcus aureus*. Expert Rev anti-infective Therapy. 2015;13(5):605–13. 10.1586/14787210.2015.1023291. 25746414 10.1586/14787210.2015.1023291

[26] Vestweber PK, Wächter J, Planz V, Jung N, Windbergs M. The interplay of Pseudomonas aeruginosa and Staphylococcus aureus in dual-species biofilms impacts development, antibiotic resistance and virulence of biofilms in in vitro wound infection models. PLoS ONE. 2024;19(5):e0304491. 10.1371/journal.pone.0304491. eCollection 2024. 38805522 10.1371/journal.pone.0304491 PMC11132468

[27] Reigada I, San-Martin-Galindo P, Gilbert-Girard S, Chiaro J, Cerullo V, Savijoki K, et al. Surfaceome and exoproteome dynamics in dual-species *Pseudomonas aeruginosa* and *Staphylococcus aureus* biofilms. Front Microbiol. 2021;2:672975. 10.3389/fmicb.2021.672975. 10.3389/fmicb.2021.672975 PMC8267900 34248881

[28] Qin Z, Yang L, Qu D, Molin S, Tolker-Nielsen T. *Pseudomonas aeruginosa* extracellular products inhibit Staphylococcal growth, and disrupt established biofilms produced by *Staphylococcus epidermidis*. Microbiology. 2017;155(7):2148–56. 10.1099/mic.0.028001-0. 10.1099/mic.0.028001-0 19389780

[29] Thierbach S, Wienhold M, Fetzner S, Hennecke U. Synthesis and biological activity of methylated derivatives of the *Pseudomonas metabolites* HHQ, HQNO and PQS. Beilstein J Org Chem. 2019;15(1):187–93. 10.3762/bjoc.15.18. 30745993 10.3762/bjoc.15.18 PMC6350858

[30] Naik P, Pandey S, Gagan S, Biswas S, Joseph J. Virulence factors in multidrug (MDR) and Pan-drug resistant (XDR) *Pseudomonas aeruginosa*: a cross-sectional study of isolates recovered from ocular infections in a high-incidence setting in Southern India. J Ophthalmic Inflamm Infect. 2021;11:1–11. 10.1186/s12348-021-00268-w. 34585284 10.1186/s12348-021-00268-w PMC8479063

[31] Edward EA, El Shehawy MR, Abouelfetouh A, Aboulmagd E. Prevalence of different virulence factors and their association with antimicrobial resistance among *Pseudomonas aeruginosa* clinical isolates from Egypt. BMC Microbiol. 2023;23(1):161. 10.1186/s12866-023-02897-8. 37270502 10.1186/s12866-023-02897-8 PMC10239191

[32] Sendra E, Fernández-Muñoz A, Zamorano L, Oliver A, Horcajada JP, Juan C, et al. Impact of multidrug resistance on the virulence and fitness of *Pseudomonas aeruginosa*: a Microbiological and clinical perspective. Infection. 2024;52(4):1235–68. 10.1007/s15010-024-02313-x. 38954392 10.1007/s15010-024-02313-x PMC11289218

[33] Hoffman LR, Déziel E, d’Argenio DA, Lépine F, Emerson J, McNamara S, et al. Selection for *Staphylococcus aureus* small-colony variants due to growth in the presence of *Pseudomonas aeruginosa*. Proc Natl Acad Sci USA. 2006;103(52):19890–5. 10.1073/pnas.0606756104. 17172450 10.1073/pnas.0606756104 PMC1750898

[34] Ahlgren HG, Benedetti A, Landry JS, Bernier J, Matouk E, Radzioch D, et al. Clinical outcomes associated with *Staphylococcus aureus* and *Pseudomonas aeruginosa* airway infections in adult cystic fibrosis patients. BMC Pulm Med. 2015;15:1–6. 10.1186/s12890-015-0062-7. 26093634 10.1186/s12890-015-0062-7 PMC4475617

[35] Limoli DH, Yang J, Khansaheb M, Helfman B, Peng L, Stecenko A, Goldberg JB. Staphylococcus aureus and Pseudomonas aeruginosa co-infection is associated with cystic fibrosis-related diabetes and poor clinical outcomes. Eur J Clin Microbiol Infect Dis. 2016;35(6):947–53. 10.1007/s10096-016-2621-0. 26993289 10.1007/s10096-016-2621-0

[36] Tognon M, Köhler T, Luscher A, Van Delden C. Transcriptional profiling of *Pseudomonas aeruginosa* and *Staphylococcus aureus* during in vitro co-culture. BMC Genomics. 2019;20:1–15. 10.1186/s12864-018-5398-y. 30630428 10.1186/s12864-018-5398-y PMC6327441

[37] Fuchs S, Pané-Farré J, Kohler C, Hecker M, Engelmann S. Anaerobic gene expression in *Staphylococcus aureus*. J Bacterial. 2007;189(11):4275–89. 10.1128/JB.00081-07. 10.1128/JB.00081-07 PMC1913399 17384184

[38] Petrova OE, Schurr JR, Schurr MJ, Sauer K. Microcolony formation by the opportunistic pathogen *Pseudomonas aeruginosa* requires pyruvate and pyruvate fermentation. Mol Microbiol. 2012;86(4):819–35. 10.1111/mmi.12018. 22931250 10.1111/mmi.12018 PMC3527662

[39] Goodwine J, Gil J, Doiron A, Valdes J, Solis M, Higa A, Davis S, Sauer K. Pyruvate-depleting conditions induce biofilm dispersion and enhance the efficacy of antibiotics in killing biofilms in vitro and in vivo. Sci Rep. 2019;9(1):3763. 10.1038/s41598-019-40378-z. 30842579 10.1038/s41598-019-40378-z PMC6403282

