# Supplementary material for: Assessment of the effect of culture components of clinical Pseudomonas aeruginosa isolates on vancomycin eradication of Staphylococcus aureus mature biofilms
Source: BMC Res Notes. 2026 Mar 10;19:162. doi: 10.1186/s13104-026-07718-5 (PMC13063584; doi:10.1186/s13104-026-07718-5)
Supplement: Supplementary file 1 — Supplementary Material 1. [file 13104_2026_7718_MOESM1_ESM.docx]

**Fig S1: Antimicrobial susceptibility testing results of the 5 tested MRSA isolates.** Summary of the susceptibility results as determined by Phoenix BD automated system for identification and antimicrobial susceptibility testing.

**Fig S2**: **Antimicrobial susceptibility testing results of the 5 tested MSSA isolates.** Summary of the susceptibility results as determined by Phoenix BD automated system for identification and antimicrobial susceptibility testing.

**Fig S3: Antimicrobial susceptibility testing results of the 5 tested P. aeruginosa isolates.** Summary of the susceptibility results as determined by Phoenix BD automated system for identification and antimicrobial susceptibility testing.


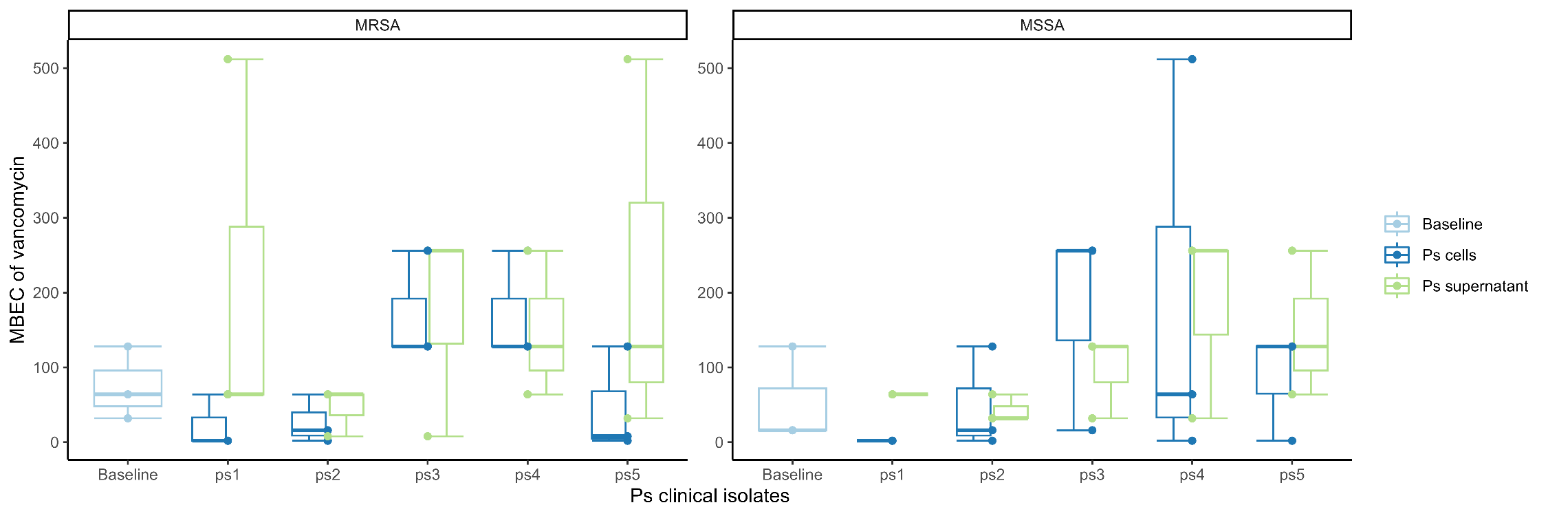


**Fig** **S4: Boxplots showing the effect of 5 *P. aeruginosa* clinical isolates on MBEC of vancomycin against MRSRA and MSSA isolates.** The thick horizontal line in each boxplot represents the median value of MBEC while the upper and lower sides of the box are q3 and q1, respectively. The upper and lower whiskers show the maximum and minimum value of MBEC. MBEC: Minimal biofilm eradication concentration. ps1, ps2, ps3, ps4, ps5 correspond to P1, P2, P3, P4, and P5, respectively.

**
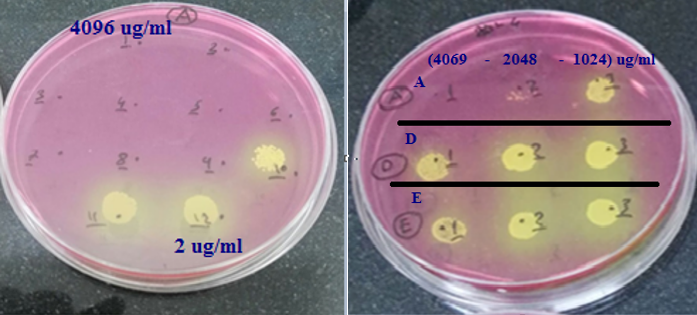
**

**Fig S5: Drop plate technique for determination of MBEC of vancomycin for MSSA.6 in the planktonic phase of biofilm at baseline (left) and in the sessile phase (right) at baseline and after interaction with *P. aeruginosa* (P2) cells/supernatant.** On the left: Numbers on the plate (1-12) indicate drops from wells of the MTP containing two-fold serial dilutions of vancomycin starting from 4096 μg /ml to 2μg/ml. Baseline MBEC of MSSA.6 planktonic phase corresponded to drop number 9 from the well containing 16 μg/ml of vancomycin. On the right: Numbers on the plate (1-3) indicate drops from wells of the MTP containing two-fold serial dilutions of vancomycin starting from 4096 μg /ml to 1024μg/ml. (**A**) Baseline MBEC of MSSA.6 corresponded to drop number 1 from the well containing 4096 μg/ml. (**D**) MBEC of MSSA.6 after disruption with P2 freshly prepared culture was > 4096 μg/ml. (**E**) MBEC of MSSA.6 after disruption with P2 cell free culture supernatant was > 4096 μg/ml.

**Table S1. Primers sequences for *ldh*, and *adh* genes.**

| **Sequence Name** | **Sequence 5'-3'** |
| --- | --- |
| ***ldh* forward primer** | TGCTGGTGCTGCACAAAAACC |
| ***ldh* reverse primer** | CGTGGCGCAACATCGAACG |
| ***adh* forward primer** | TGTGGCGTTTGTCATACCGA |
| ***adh* reverse primer** | ATAGAAGACGCTGCTGCTGG |

**Table S2. The effect of biofilm disruption with *P. aeruginosa* (strain P1) cell suspension and cell free culture supernatant on the viable cell counts of sessile phase cells of MRSA biofilms**

| ***S. aureus* ID** | **Baseline cell counts (CFU/ml)** | **After P1 cell suspension** | | **After P1 supernatant** | |
| --- | --- | --- | --- | --- | --- |
|  |  | **Cell counts (CFU/ml)** | **Log_10_ reduction** | **Cell counts (CFU/ml)** | **Log_10_ reduction** |
| MRSA. 1 | 2.00 × 10⁸ | 2.50 × 10⁶ | 1.90 | 1.50 × 10⁸ | 0.12 |
| MRSA. 17 | 1.35 × 10⁸ | 8.50 × 10⁶ | 1.20 | 8.00 × 10⁷ | 0.23 |
| MRSA. 18 | 1.40 × 10⁸ | 3.00 × 10⁷ | 0.67 | 1.10 × 10⁸ | 0.10 |
| MRSA. 21 | 1.45 × 10⁸ | 2.00 × 10⁷ | 0.86 | 8.00 × 10⁷ | 0.26 |
| MRSA. 22 | 3.20 × 10⁸ | 8.00 × 10⁶ | 1.60 | 8.00 × 10⁷ | 0.60 |
| **Overall** |  |  |  |  |  |
| **Mean** |  |  | 1.25 |  | 0.26 |
| **SD** |  |  | 0.510 |  | 0.200 |

Log₁₀ reduction was calculated as log₁₀(CFU/mL at baseline) − log₁₀(CFU/mL after treatment). A value of 0 indicates no reduction in viable cell counts.

**Table S3: The Effect of biofilm disruption with *P. aeruginosa* (strain P1) cell suspension and cell free culture supernatant on the viable cell counts of sessile phase cells of MSSA biofilms**

| ***S. aureus* ID** | **Baseline cell counts (CFU/ml)** | **After P1 cell suspension** | | **After P1 supernatant** | |
| --- | --- | --- | --- | --- | --- |
|  |  | **Cell counts (CFU/ml)** | **Log_10_ reduction** | **Cell counts (CFU/ml)** | **Log_10_ reduction** |
| MSSA. 6 | 3.5 × 10⁸ | 1.40 × 10⁸ | 0.40 | 1.50 × 10⁸ | 0.37 |
| MSSA. 7 | 8.2 × 10⁸ | 4.0 × 10⁷ | 1.31 | 1.00 × 10⁸ | 0.91 |
| MSSA. 8 | 6.5 × 10⁸ | 3.0 × 10⁷ | 1.34 | 1.10 × 10⁸ | 0.77 |
| MSSA. 9 | 4.5 × 10⁸ | 5.0 × 10⁷ | 0.95 | 1.00 × 10⁸ | 0.65 |
| MSSA. 10 | 2.5 × 10⁹ | 3.0 × 10⁷ | 1.92 | 5.0 × 10⁷ | 1.70 |
| **Overall** |  |  |  |  |  |
| **Mean** |  |  | 1.18 |  | 0.88 |
| **SD** |  |  | 0.560 |  | 0.499 |

Log₁₀ reduction was calculated as log₁₀(CFU/mL at baseline) − log₁₀(CFU/mL after treatment). A value of 0 indicates no reduction in viable cell counts.

**Table S4. MBEC of vancomycin on *S. aureus* in the planktonic and sessile phases of biofilms, before and after interaction with P1 cell suspension and cell free culture supernatant.**

| ***S. aureus* strain number** | **Baseline MBEC in planktonic phase** | **MBEC after interaction in planktonic phase** | | **Baseline MBEC in sessile phase** | **MBEC after interaction in sessile phase** | |
| --- | --- | --- | --- | --- | --- | --- |
|  |  | **P1**  **Cells** | **P1 supernatant** |  | **P1**  **cells** | **P1 supernatant** |
| **MSSA. 6** | 16 ug/ml | < 2 ug/ml | 64 ug/ml | 4096 ug/ml | 4096 ug/ml | > 4096 ug/ml |
| **MSSA. 8** | 16 ug/ml | < 2 ug/ml | 64 ug/ml | 4096 ug/ml | 4096 ug/ml | > 4096 ug/ml |
| **MSSA. 9** | 128 ug/ml | < 2 ug/ml | 64 ug/ml | > 4096 ug/ml | > 4096 ug/ml | > 4096 ug/ml |
| **MRSA.17** | 128 ug/ml | 64 ug/ml | 64 ug/ml | > 4096 ug/ml | > 4096 ug/ml | > 4096 ug/ml |
| **MRSA. 18** | 32 ug/ml | < 2 u/ml | 64 ug/ml | 4096 ug/ml | > 4096 ug/ml | > 4096 ug/ml |
| **MRSA. 21** | 64 ug/ml | < 2 ug/ml | 512 ug/ml | > 4096 ug/ml | > 4096 ug/ml | > 4096 ug/ml |

**Table S5. MBEC of vancomycin on *S. aureus* in planktonic and sessile phases of biofilms, before and after interaction with P2 cell suspension and cell free culture supernatant.**

| ***S. aureus* strain number** | **Baseline MBEC in planktonic phase** | **MBEC after interaction in planktonic phase** | | **Baseline MBEC in sessile phase** | **MBEC after interaction in sessile phase** | |
| --- | --- | --- | --- | --- | --- | --- |
|  |  | **P2**  **Cells** | **P2 supernatant** |  | **P2**  **cells** | **P2 supernatant** |
| **MSSA. 6** | 16 ug/ml | 128 ug/ml | 32 ug/ml | 4096 ug/ml | > 4096 ug/ml | > 4096 ug/ml |
| **MSSA. 8** | 16 ug/ml | < 2 ug/ml | 64 ug/ml | 4096 ug/ml | > 4096 ug/ml | > 4096 ug/ml |
| **MSSA. 9** | 128 ug/ml | 16 ug/ml | 32 ug/ml | > 4096 ug/ml | > 4096 ug/ml | > 4096 ug/ml |
| **MRSA. 17** | 128 ug/ml | 64 ug/ml | 64 ug/ml | > 4096 ug/ml | > 4096 ug/ml | > 4096 ug/ml |
| **MRSA. 18** | 32 ug/ml | 16 ug/ml | 64 ug/ml | 4096 ug/ml | > 4096 ug/ml | > 4096 ug/ml |
| **MRSA. 21** | 64 ug/ml | < 2 ug/ml | 8 ug/ml | > 4096 ug/ml | > 4096 ug/ml | > 4096 ug/ml |

**Table S6. MBEC of vancomycin on *S. aureus* in planktonic and sessile phases of biofilms, before and after interaction with *P.*3 cell suspension and cell free culture supernatant.**

| ***S. aureus* strain number** | **Baseline MBEC in planktonic phase** | **MBEC after interaction in planktonic phase** | | **Baseline MBEC in sessile phase** | **MBEC after interaction in sessile phase** | |
| --- | --- | --- | --- | --- | --- | --- |
|  |  | **P3**  **Cells** | **P3 supernatant** |  | **P3**  **cells** | **P3 supernatant** |
| **MSSA. 6** | 16 ug/ml | 256 ug/ml | 128 ug/ml | 4096 ug/ml | > 4096 ug/ml | > 4096 ug/ml |
| **MSSA. 8** | 16 ug/ml | 16 ug/ml | 128 ug/ml | 4096 ug/ml | > 4096 ug/ml | > 4096 ug/ml |
| **MSSA. 9** | 128 ug/ml | 256 ug/ml | 32 ug/ml | > 4096 ug/ml | > 4096 ug/ml | > 4096 ug/ml |
| **MRSA. 17** | 128 ug/ml | 128 ug/ml | 256 ug/ml | > 4096 ug/ml | > 4096 ug/ml | > 4096 ug/ml |
| **MRSA. 18** | 32 ug/ml | 128 ug/ml | 256 ug/ml | 4096 ug/ml | > 4096 ug/ml | > 4096 ug/ml |
| **MRSA. 21** | 64 ug/ml | 256 ug/ml | 8 ug/ml | > 4096 ug/ml | > 4096 ug/ml | > 4096 ug/ml |

**Table S7. MBEC of vancomycin on *S. aureus* in planktonic and sessile phases of biofilms, before and after interaction with *P*.4 cell suspension and cell free culture supernatant.**

| ***S. aureus* strain number** | **Baseline MBEC in planktonic phase** | **MBEC after interaction in planktonic phase** | | **Baseline MBEC in sessile phase** | **MBEC after interaction in sessile phase** | |
| --- | --- | --- | --- | --- | --- | --- |
|  |  | **P4**  **Cells** | **P4 supernatant** |  | **P4**  **cells** | **P4 supernatant** |
| **MSSA. 6** | 16 ug/ml | 512 ug/ml | 256 ug/ml | 4096 ug/ml | > 4096 ug/ml | 2048 ug/ml |
| **MSSA. 8** | 16 ug/ml | <2 ug/ml | 256 ug/ml | 4096 ug/ml | > 4096 ug/ml | > 4096 ug/ml |
| **MSSA. 9** | 128 ug/ml | 64 ug/ml | 32 ug/ml | > 4096 ug/ml | > 4096 ug/ml | > 4096 ug/ml |
| **MRSA. 17** | 128 ug/ml | 128 ug/ml | 128 ug/ml | > 4096 ug/ml | > 4096 ug/ml | > 4096 ug/ml |
| **MRSA. 18** | 32 ug/ml | 256 ug/ml | 256 ug/ml | 4096 ug/ml | > 4096 ug/ml | > 4096 ug/ml |
| **MRSA. 21** | 64 ug/ml | 128 ug/ml | 64 ug/ml | > 4096 ug/ml | > 4096 ug/ml | > 4096 ug/ml |

**Table S8. MBEC of vancomycin on *Staphylococcus aureus* in planktonic and sessile phases of biofilms, before and after interaction with *P.*5 cell suspension and cell free culture supernatant.**

| ***S. aureus* strain number** | **Baseline MBEC in planktonic phase** | **MBEC after interaction in planktonic phase** | | **Baseline MBEC in sessile phase** | **MBEC after interaction in sessile phase** | |
| --- | --- | --- | --- | --- | --- | --- |
|  |  | **P5**  **Cells** | **P5 supernatant** |  | **P5**  **cells** | **P5 supernatant** |
| **MSSA. 6** | 16 ug/ml | 128 ug/ml | 64 ug/ml | 4096 ug/ml | > 4096 ug/ml | > 4096 ug/ml |
| **MSSA. 8** | 16 ug/ml | <2 ug/ml | 128 ug/ml | 4096 ug/ml | > 4096 ug/ml | 4096 ug/ml |
| **MSSA. 9** | 128 ug/ml | 128 ug/ml | 256 ug/ml | > 4096 ug/ml | > 4096 ug/ml | > 4096 ug/ml |
| **MRSA. 17** | 128 ug/ml | <2 ug/ml | 32 ug/ml | > 4096 ug/ml | > 4096 ug/ml | > 4096 ug/ml |
| **MRSA. 18** | 32 ug/ml | 8 ug/ml | 128 ug/ml | 4096 ug/ml | > 4096 ug/ml | > 4096 ug/ml |
| **MRSA. 21** | 64 ug/ml | 128 ug/ml | 512 ug/ml | > 4096 ug/ml | > 4096 ug/ml | > 4096 ug/ml |

**Table S9. Changes in the mean MBEC values of vancomycin for *S. aureus* biofilm cells (planktonic) after treatment with *P. aeruginosa* (P1 – P5) cells and supernatants**

| ***S. aureus* studied groups** | **Baseline**  ***Ln MBEC** | **Effect of *P. aeruginosa* supernatant on *Ln MBEC** | **Effect of *P. aeruginosa* cells on *Ln MBEC** |
| --- | --- | --- | --- |
| **All *S. aureus***  Mean & SD  t-ratio  (*p*) | (n = 6)  3.81 ± 0.96 | (n = 30)  4.44 ± 1.04  1.845  (.069) | (n = 30)  3.23 ± 2.04  1.709  (.092) |
| **MRSA**  Mean & SD  t-ratio  (*p*) | (n = 3)  4.16 ± 0.69 | (n = 15)  4.48 ± 1.28  0.671  (.504) | (n = 15)  3.42 ± 1.93  1.53  (.258) |
| **MSSA**  Mean & SD  t-ratio  (*p*) | (n = 3)  3.47 ± 1.20 | (n = 15)  4.39 ± 0.77  1.92  (.118) | (n = 15)  3.05 ± 2.19  0.863  (.391) |

Ln MBEC (natural logarithm of MBEC values of vancomycin on *S. aureus* tested isolates), t = model-based t-ratio from linear mixed-effects analysis, (p) = Holm-adjusted *p*-value. No statistically significant differences were observed (adjusted p > 0.05).
